# Pickering Emulsions Based on Wax and Halloysite Nanotubes: An Ecofriendly Protocol for the Treatment of Archeological Woods

**DOI:** 10.1021/acsami.0c20443

**Published:** 2020-12-30

**Authors:** Lorenzo Lisuzzo, Theodore Hueckel, Giuseppe Cavallaro, Stefano Sacanna, Giuseppe Lazzara

**Affiliations:** † Molecular Design Institute, Department of Chemistry, New York University, 29 Washington Place, New York, New York 10003, United States; ‡ Department of Physics and Chemistry, University of Palermo, Viale delle Scienze, pad. 17, Palermo 90128, Italy

**Keywords:** halloysite, Pickering emulsion, wood, nanocomposite, wax, nanotubes

## Abstract

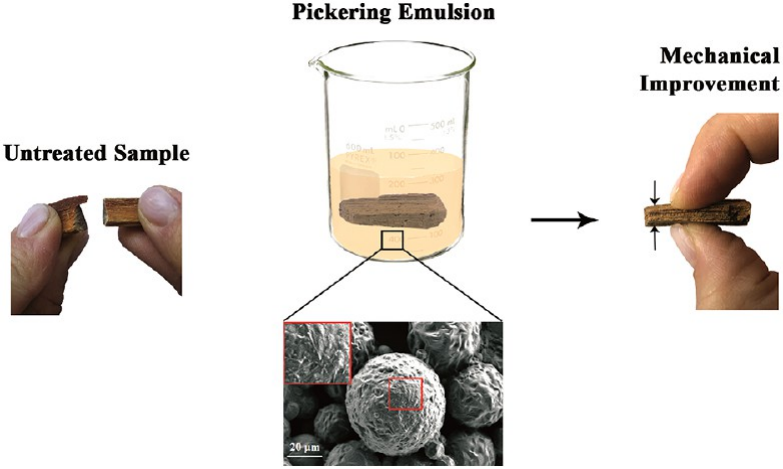

A novel green protocol for the consolidation and protection of waterlogged archeological woods with wax microparticles has been designed. First, we focused on the development of halloysite nanotubes (HNTs) based Pickering emulsions using wax as the inner phase of the oil-in-water droplets. The optimization of the preparation strategy was supported by both optical microscopy and scanning electron microscopy, which allowed us to show the morphological features of the prepared hybrid systems and their structural properties, i.e., the distribution of the clay at the interface. Also, the dependence of the overall dimensions of the prepared systems on the halloysite content was demonstrated. Microdifferential scanning calorimetry (μ-DSC) was conducted in order to assess whether the thermal properties of the wax are affected after its interaction with HNTs. Then, the Pickering emulsions were employed for the treatment of waterlogged wooden samples. Compared to the archeological woods treated with pure wax, the addition of nanotubes induced a remarkable improvement in the mechanical performance in terms of stiffness and flexural strength. The proposed protocol is environmentally friendly since water is the only solvent used throughout the entire procedure, even if wax is vehiculated into the pores at room temperature. As a consequence, the design of wax/halloysite Pickering emulsions represents a promising strategy for the preservation of wooden artworks, and it has a great potential to be scaled up, thus becoming also exploitable for the treatments of shipwrecks of large size.

## 1 Introduction

In recent years, the consolidation and protection of historical artifacts have attracted the interest of many researchers due to their great importance as evidence of the past but also for the difficulty to fulfill this extremely complex purpose.^1^ Within this field, the conservation of waterlogged archeological woods is a critical issue.^2^ After several centuries in marine or anoxy and humid environments, where the wooden structures are preserved by low temperatures and low amounts of oxygen, preventing their collapse upon recovery and drying represents the most challenging task.^3^ Under water, the wood loses mechanical resistance because of the degradation of lignocellulosic polysaccharides and lignin carried out by bacteria and fungi.^4,5^ Consequently, the main concern related to the drying process is the loss of the original structure and, therefore, the loss of the historical artwork itself.^6^ New protocols have been developed for the conservation of cultural heritage.^7,8^ Colloidal engineering has become a deeply investigated research domain, and many synthetic routes have been proposed for chemists and materials scientists.^9−12^ Innovative technologies have been developed for the design of smart materials to be used in many application fields.^13−16^

For instance, different polymers have been studied for the consolidation of wooden cultural heritage, and, among them, colophonies, poly(ethylene) glycols, poly(propylene) glycols, cellulose ethers, sugars, and epoxy resins are a few examples.^17−21^ Indeed, the use of colophony was investigated because it presents several advantages for the stabilization of wood in terms of dimensions, shape, and maintenance over time.^22^ However, similarly to the other polymers, highly volatile solvents are needed for the wood impregnation because they optimize the polymeric transport and filling within the wood channels.^23^ This point represents a critical issue for the environmental impact and possibility to scale up the proposed procedures. Also, chitosan can be used for the treatment of wooden artifacts, but it requires acetic acid for its solubilization and the presence of inorganic salts (e.g., potassium nitrite) for depolymerization.^24^

Nowadays, the most common procedures for the treatment of archeological wood from ancient shipwrecks deal with the use of PEGs with different molecular weights.^25^ This method allowed the attainment of high efficiency in the short term, but it presents some detrimental consequences in the long term.^26,27^ According to the literature, the use of PEG enhances the degradation of the treated samples by increasing the acidity inside the wooden structure.^28^ This is due to the production over time of acid compounds resulting from the interactions between the polymeric chains of PEGs and sulfate or iron species, further increasing the degradation extent.^29^ Among others, these effects led to the damage of the *Vasa*, a Swedish warship from the 17th century.^30,31^

To overcome this, the use of sucrose was considered a good alternative for the conservation of archeological woods, until it was observed that its long-term result is a brown sticky film on the surface of the treated samples.^32^ Therefore, many questions are still to be solved.

Lately, the remarkable advance of nanotechnology highlighted the importance of inorganic nanoparticles, such as nanoclays, for the conservation of artworks formed by lignin and cellulose.^33,34^ These systems are very promising nanofillers, and their encapsulation inside channels and pores can effectively improve both the mechanical resistance and thermal stability of waterlogged samples.^35^ Among them, halloysite nanotubes (HNTs) are naturally occurring aluminosilicates with unit formula Al_2_Si_2_O_5_(OH)_4_·*n*H_2_O, where *n* is the number of interlayer water molecules and can be *n* = 0 and *n* = 2 for the hydrate or dehydrate nanoclay with a resulting interlayer distance of 0.7 and 1 nm, respectively.^36,37^ HNTs’ most peculiar feature is their typical hollow tubular shape and their dimensions, which depend on the deposit from which the raw material is extracted. Indeed, the external diameter can vary from 50 to 100 nm and the internal diameter from 15 to 50 nm, and their length is highly polydispersed.^38,39^ Moreover, the inner lumen of nanotubes represents an encapsulation site that can be loaded with oppositely charged guest molecules and bioactive species.^40^ Thus, they can act as nanocontainers and nanocarriers.^41−43^ Also, halloysite showed ecosustainability and nontoxicity in several in vitro and in vivo studies.^44−46^ Due to all these characteristics, numerous advanced applications have been discovered for this clay, e.g., biomedical and health science, environmental remediation and catalysis, food packaging, cosmetics, and pharmaceuticals.^47−59^ The use of halloysite for the conservation of cultural heritage holds a certain importance. For instance, since the production of acids makes PEGs harmful for the wooden structure, literature reports a procedure for the deacidification of previously treated archeological woods by using Ca(OH)_2_-loaded HNTs as nanofillers with antiacid activity. In this case, the combination of the polymer with nanotubes was revealed to be efficient for the protection and the deacidifying consolidation of the wooden artworks.^60^ Also, beeswax/HNTs and esterified colophony/HNTs nanocomposites were designed as consolidants for waterlogged woods.^61^

Notwithstanding their promising efficiencies, these protocols need acetone as an organic volatile solvent. As reported above, these aspects present some crucial limitations concerning the sustainability of the methods and the possibility to be exploited for the treatment of shipwrecks of large dimensions. In light of this, the aim of this work is the design of a novel material for the long-term preservation of waterlogged archeological wood in order to overcome the constraints related to the use of PEGs or volatile organic solvents. It is well-known, since the pioneering studies of Ramsden and Pickering, that emulsions can be stabilized by interfacially active solid particles which can act as emulsifiers by surrounding the oil-in-water droplet.^62−64^ Halloysite is a good and attractive candidate for this purpose.^65−67^ With this in mind, here we proposed a new protocol for the preparation of halloysite-based Pickering emulsions using wax as the inner phase of the oil-in-water droplets. Although it is soluble in different organic solvents, we exploited the peculiar thermal properties of wax (i.e., its melting transition) in order to optimize its interaction with the nanoclay in water. Since pure melted waxes do not penetrate deep into the wood and they remain on the surface, the choice to use paraffin as the inner core of the Pickering emulsions is strategic for its transport and encapsulation within the wooden structures.^68^ It is noteworthy that water is the only solvent used in the whole consolidation procedure, and, in conclusion, the combination of paraffin and halloysite nanotubes was revealed as an efficient and environmentally friendly strategy with great potential to be scaled up, representing the starting point for the design of a green protocol to preserve wooden structures of historical and cultural value.

## 2 Materials and Methods

### 2.1 Materials

Halloysite nanotubes (HNTs) are a gift from I-Minerals Inc. mined in the geological deposit of Latah County with the physicochemical properties detailed elsewhere.^69^ Paraffin wax (melting point 45–58 °C) was purchased by Sigma-Aldrich. Acetone (99.5%, 0.791 g cm^3^) is a Panreac reagent. The waterlogged archeological woods are from the ship Chretienne C (II century, BC) discovered over the coast of Provence and kindly provided by Prof. Patrice Pomey from CNRS, Universite′ de Provence (France). On the basis of the procedure of the Italian standard (UNI 11205:2007), the archeological woods were identified as *Pinus pinaster*.^70^

### 2.2 Pickering Emulsions Preparation

HNTs-stabilized Pickering emulsions were prepared by mixing the nanotubes and molten paraffin wax in hot water. Briefly, 0.1 g of solid wax is added to 10 mL of deionized water at 80 °C under magnetic stirring. When the wax is fully molten and emulsified, halloysite nanotubes are added, and the sample sonicated for 5 min. The resulting Pickering emulsion is left to equilibrate under stirring (∼500 rpm) for 30 min at 80 °C before it is quenched in an ice bath. The microparticles are washed three times in deionized water by centrifugation to remove the excess of HNTs. Finally, they are redispersed in the same amount of water. This procedure was used with different amounts of clay nanoparticles, ranging from 0.05 to 1% w/w.

### 2.3 Treatment of Waterlogged Archeological Woods

The waterlogged archeological wood was consolidated through the immersion method as described elsewhere.^60^ Dispersions of wax/HNTs Pickering emulsions in water at different compositions of clay were employed as consolidant mixtures. The archeological woods were kept in the paraffin/nanoclay mixtures under stirring for 3 days. The efficiency of the treatment was evaluated by determining the mechanical performances of the treated wood samples. Impregnation from paraffin/acetone dispersion was applied only to evaluate the pure wax consolidation efficiency, without any clay addition.

### 2.4 Characterization

Optical bright field microscopy images were acquired using a Leica DMI3000 inverted microscope equipped with DIC optics and high-resolution CMOS camera (Hamamatsu ORCA Flash4.0 sCMOS). The morphological features and organization at a nanometric scale of wax/HNTs Pickering emulsions were studied by scanning electron microscopy, which was conducted using a MERLIN (Carl Zeiss) field emission SEM. To avoid charging under the electron beam, 200 μL of each sample was dried on SEM stubs and sputtered-coated with a 3 nm layer of iridium in argon by using a Cressington 208HR Sputter Coater. A statistical analysis on the dimensions of the prepared samples as a function of the clay content was conducted using ImageJ as software.^71^ To do so, the diameter of all the Pickering emulsions in the SEM images with the lowest magnification (more particles) for every sample was measured, and the average size was calculated. Micro differential scanning calorimetry (μ-DSC) analysis was performed by using a Setaram DSC III apparatus. Each sample was treated according to a specific temperature program, following three cycles of heating intermediated by three cycles of cooling. In particular, samples were heated from 5 to 85 °C with the same scale rate of 1 °C min^–1^ for all the heating cycles and cooled from 85° to 5 °C with a variable scale rate: 1 °C min^–1^ for the first cycle, 0.5 °C min^–1^ for the second cycle, and 0.3 °C min^–1^ for the last cycle. All the heating curves are similar to each other. The stainless steel (1 cm^3^) sample cell was filled with ca. 500 mg of the aqueous dispersion and the reference cell with the corresponding amount of water. The calibration was performed by using naphthalene. Δ*C*_*p*_ values were normalized to the wax weight fraction by considering the experimental composition of the investigated materials. Data analysis such as deconvolution of curves and nonlinear curve fitting was carried out by using Fityk as software.^72^ As concerns the consolidated archeological woods, the morphological investigation was carried out by using a Digitus (DA-70351) optical microscope. The cross sections were obtained by cutting the samples after immersion in liquid N_2_. Colorimetric analysis was conducted using a NH300 Colorimeter (3NH Shanghai Co., Ltd.). The device was calibrated using black and white plates, and CQCS3 Software was used for the data collection of L* (lightness), a* (red–green), and b* (yellow–blue). The total color difference (Δ*E*) between samples was calculated as reported in the literature by considering three different points.^73^ Dynamic mechanical measurements on dry samples were conducted by using DMA Q800 (TA Instruments). We performed three-point flexural measurements at 25 °C. The stress ramp was set at 1 MPa min^–1^. The force was applied perpendicular to the wood fibers. The analysis of the stress versus strain curves allowed us to determine the mechanical performance of the treated wood samples in terms of stress and the elongation at the breaking point as well as rigidity, which was estimated by elastic modulus. The latter was calculated by the slope of stress versus strain functions in the linear region.

## 3 Results

### 3.1 Wax/HNTs Pickering Emulsions Preparation and Characterization

The design of this new hybrid system composed of paraffin, as organic moiety, and halloysite nanotubes, as inorganic counterpart, is the result of a precise optimization of the preparation protocol.

**Figure 1:**
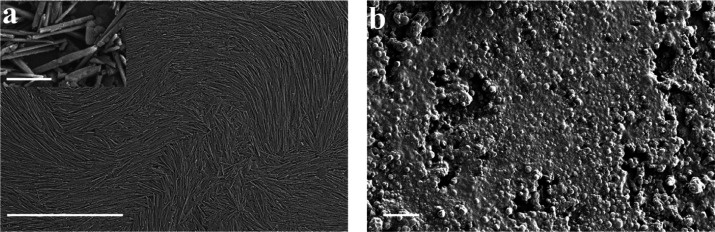
SEM image of pristine halloysite nanotubes (a) and paraffin-in-water system (b). The scale bars are 20 μm for (a) and (b) and 1 μm for the inset in (a), respectively.

At the beginning of this work, the microscopic characterization of both the starting building blocks was carried out, as reported in Figure 1.

The nanotubes, whose length is polydispersed in the 1–4 μm range, appear to have some particular orientations, and many distinct domains can be recognized in the SEM image (Figure 1a). This organization is due to the coffee ring effect investigated elsewhere.^74,75^ The peculiar hollow tubular shape of halloysite can be clearly observed in the inset in Figure 1a, which allows the determination that the external diameter is in the 50–200 nm range, as reported in the literature.^69^

Halloysite plays a major role in the formation of the Pickering emulsions. Indeed, Figure 1b shows the result of the preparation protocol carried out without any clay addition. In this case, the paraffin creates a continuous matrix due to agglomeration/coalescence and oil phase separation, as expected.

Hence, halloysite nanotubes were added to the emulsions at different concentrations, namely, 0.05, 0.1, 0.25, 0.5, 0.75, and 1% w/w. In order to prepare a significant amount of stable, homogeneous, and well-shaped Pickering emulsions, the preparation protocol was improved by a step-by-step procedure (see Figure S1).

**Figure 2:**
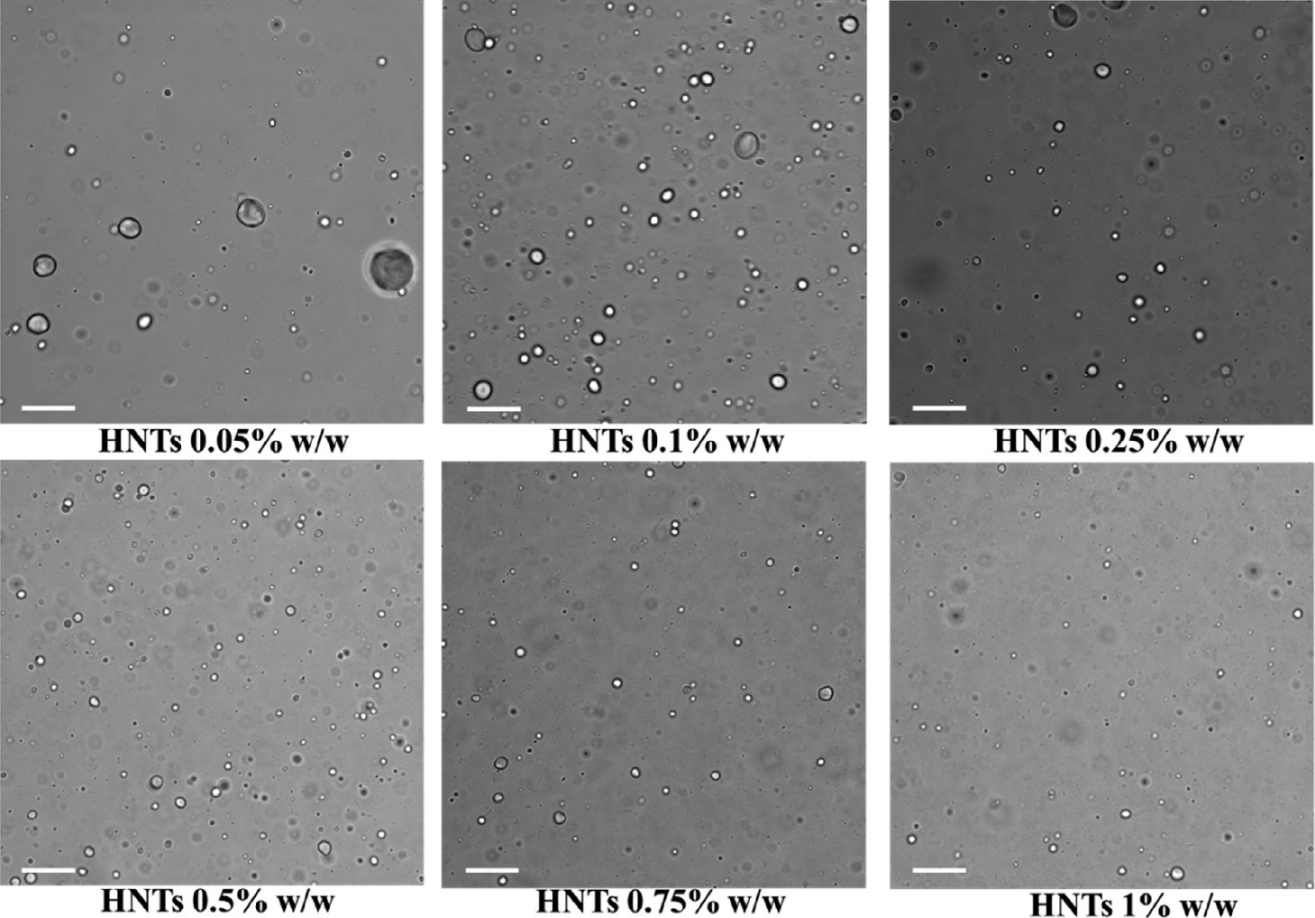
Optical images of wax/HNTs Pickering emulsions at increasing concentration of halloysite nanotubes. Scale bars are 50 μm.

Therefore, Figure 2 reports the optical images of the wax/HNTs emulsions prepared following the optimized procedure.

Herein, the presence of the spherical Pickering emulsions is observed. It is noteworthy that particles’ dimensions decrease as the halloysite concentration increases. Anyway, by using optical microscopy, it is not possible to have details on the outer shell which is composed of nanotubes.

**Figure 3:**
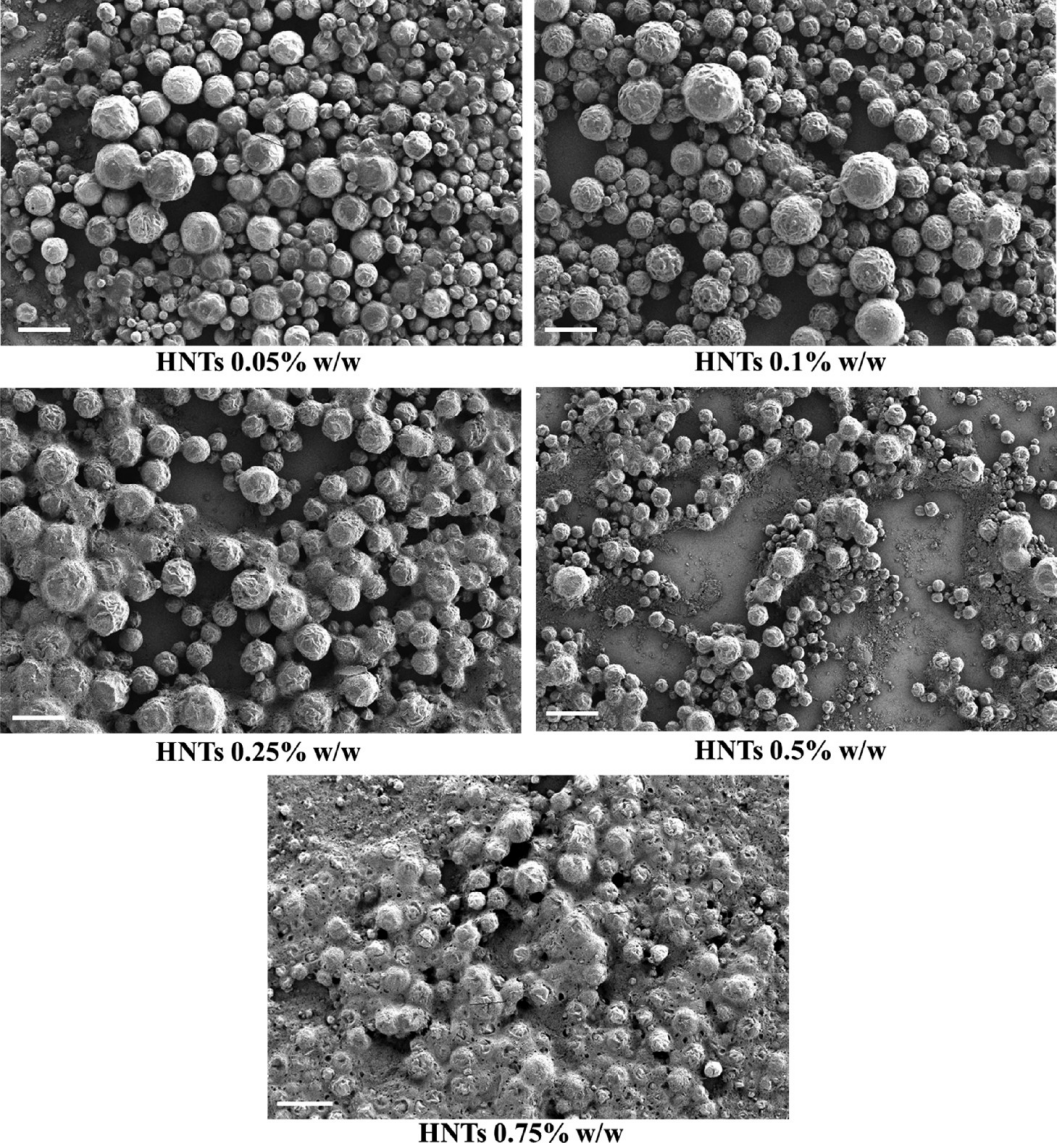
SEM images of wax/HNTs Pickering emulsions with an increasing concentration of halloysite. Scale bars are 60 μm.

For this purpose, SEM analyses were carried out in order to characterize the morphological and structural features of the Pickering emulsion with higher quality (Figure 3).

As reported in Figure 3, the development and design of the proposed protocol allowed for the preparation of a quantitative amount of paraffin/halloysite Pickering emulsions by keeping constant the concentration of wax and increasing the amount of added HNTs from 0.01 to 0.75% w/w. Nevertheless, the micrograph of the wax/HNTs Pickering emulsion with 1%w/w of halloysite concentration is reported in Figure S2. In this case, the nanoclay amount is too high to reach the emulsions preparation.

**Figure 4:**
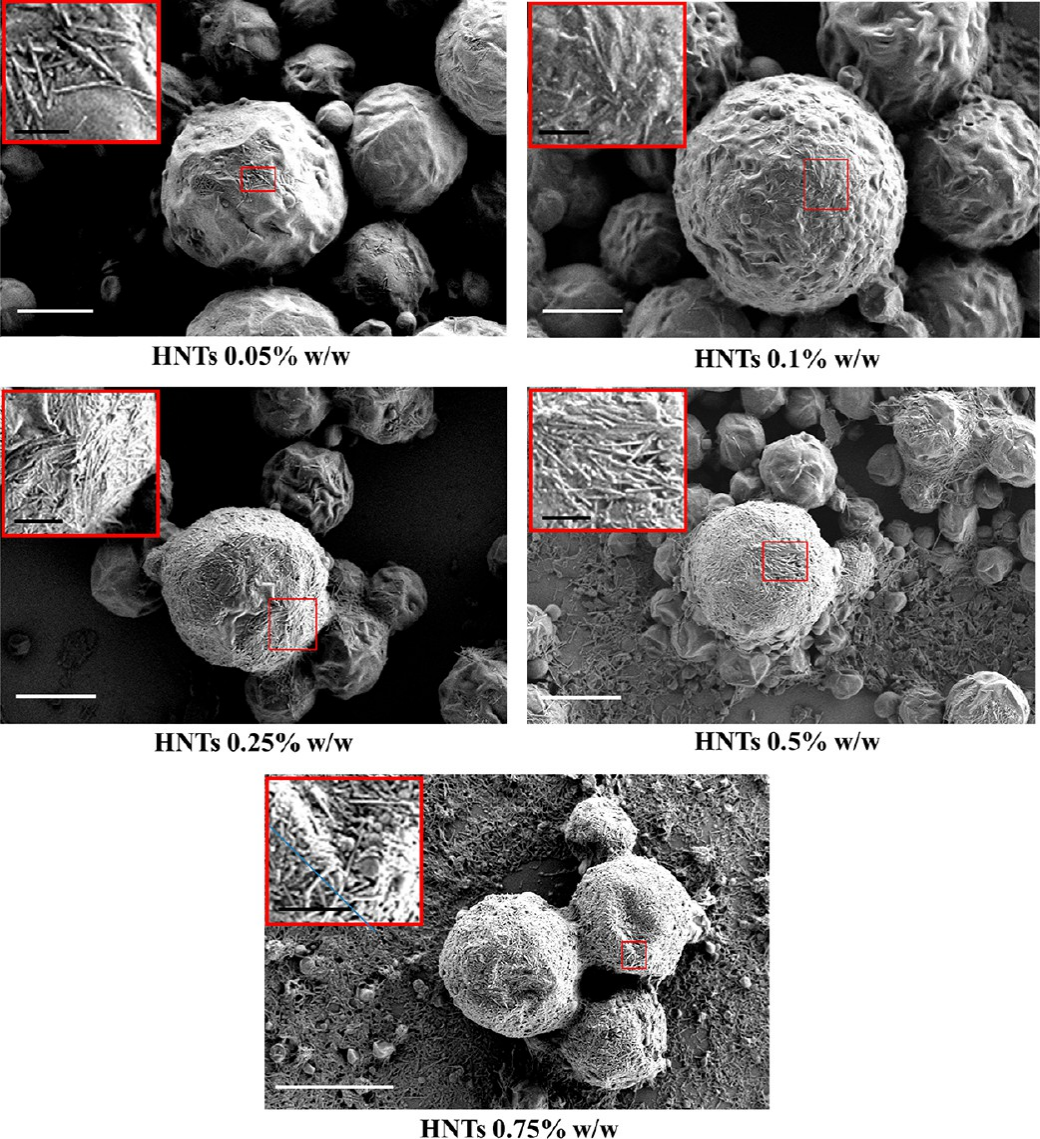
Higher magnification SEM images of wax/HNTs Pickering emulsions with an increasing concentration of halloysite. The scale bars are 20 μm for the images and 3 μm for the insets.

Some roughness at a certain extent can be observed at the outer surface of each microparticle. In order to better visualize this aspect and to have more precise insights, SEM images at a higher magnification are reported in Figure 4 for each sample.

As shown, halloysite nanotubes can be observed at the external surface of the Pickering emulsions (see insets in Figure 4). Interestingly, the coverage degree strictly depends on the amount of HNTs added during the preparation procedure, and it is higher as the concentration of nanoclay increases. This is most likely due to the particular temperature responsive behavior of paraffin that, after melting and while cooling down, catches the nanotubes in the surrounding proximity. In light of this, the quantity of halloysite stabilizing the systems is a function of its overall concentration.

**Figure 5:**
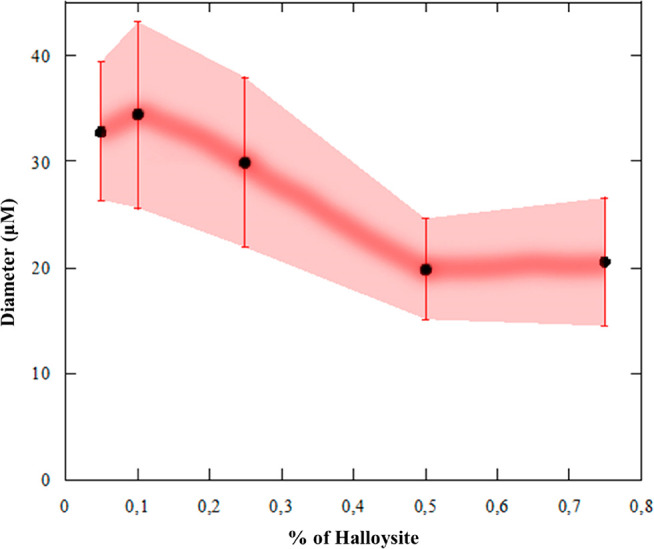
Average diameter as a function of halloysite content for wax/HNTs Pickering emulsions. Bars refer to the standard deviation of the diameter distribution.

Besides, the thermal effect on the stability of the particles was evaluated. For this purpose, the prepared Pickering emulsion system was heated up to the melting of wax. Then, it was treated again according to the preparation procedure, and stable Pickering emulsions could be reobtained. Figure S3 shows a SEM image as an example of the system recyclability. Moreover, a concentration effect of HNTs is also registered on the general dimensions of the different wax/HNTs systems. The result of a statistical study on the lowest magnification images is shown in Figure 5.

Herein, the average diameter is reported as a function of halloysite concentration. It is clear that the dimensions decreased as the nanotubes content increased, reaching a maximum of ca. 35 μm for the wax/HNTs 0.05% w/w sample and then falling to ca. 20 μm for the wax/HNTs 0.5% w/w system.

These findings are in good agreement with the literature. For instance, Pickering emulsions based on halloysite and dodecane showed a significant impact of the nanotubes concentration on the emulsions stability and average droplet sizes, with a reduction between clay concentrations of 0.05 and 0.5 wt %.^76^

**Figure 6:**
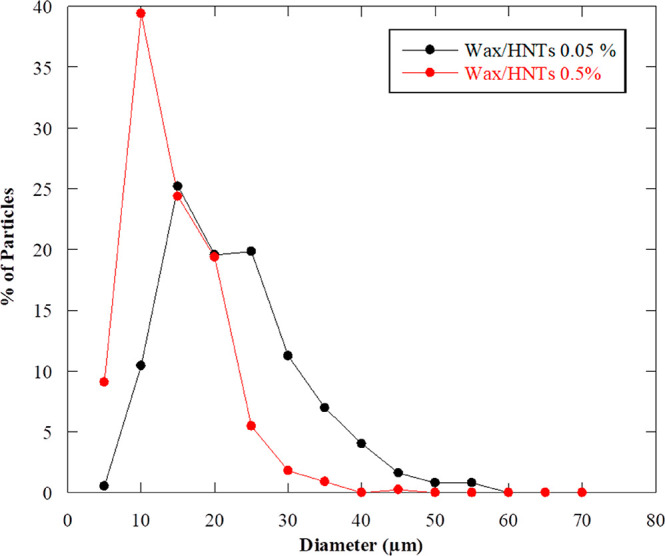
Dimensions of the Pickering emulsions for the wax/HNTs 0.05 and 0.5% w/w samples.

We also report the statistical data for both the wax/HNTs 0.05% w/w and the wax/HNTs 0.5% w/w samples after completely analyzing all the particles in each micrograph (Figure 6).

It can be observed that the curve of wax/HNTs 0.05% w/w shows a peak at higher values compared to the wax/HNTs 0.5% w/w system. For the former, 25% of particles present an average diameter of 15 μm, 20% of them possess a size of 20 μm, 20% have 25 μm as dimension, and 10% of particles show up to 30 μm in diameter. Finally, 5% of particles have an average size of 40 μm. On the other side, the wax/HNTs 0.5% w/w sample shows a narrow distribution with 85% of particles possessing an average dimension in the 0–20 μm range, having half of this population 10 μm in size. These findings confirm that the concentration of halloysite deeply affects the dimensions of the resulting Pickering emulsions, which decrease as the clay content increases, thus playing a major role in the final aspect and size distribution of such systems. As previously discussed, the adsorption of nanotubes on the external surface is enhanced by the increasing amount of halloysite in dispersion, thus leading to more stable emulsions due to the formation of a protective shell at the outer oil–water interface. Because of it, the presence of HNTs provides steric hindrance thus avoiding the coalescence of the oil droplets when the wax is still liquid and minimizing their collapse after the transition to the solid state occurs, resulting in smaller yet stable particles, as reported in the literature for different systems.^76^

### 3.2 Wax/HNTs Pickering Emulsions: Thermal Properties

**Figure 7:**
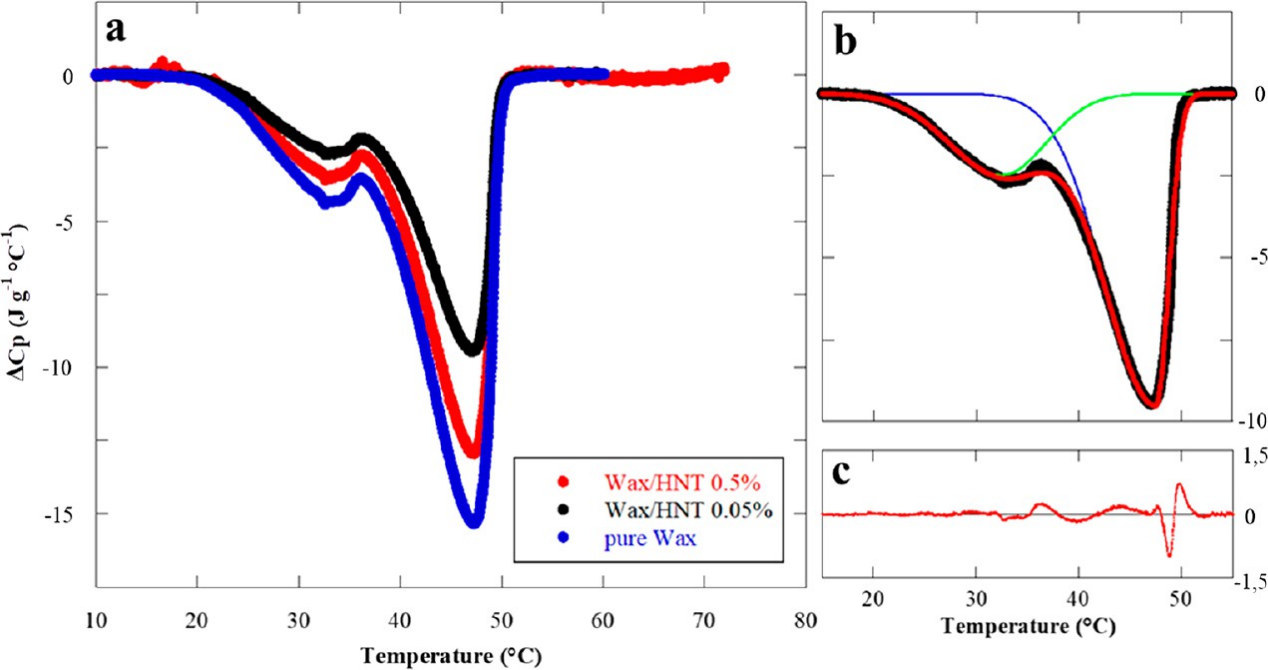
μ-DSC thermograms for pure wax, wax/HNTs 0.05% w/w, and wax/HNTs 0.5% w/w Pickering emulsions (a). DSC thermogram deconvolution for the wax/HNTs 0.05% w/w sample (b). The black line in (b) represents the experimental data, the blue and green lines represent the two curves for each peak, and the red line represents the sum of these two contributions which better fits the experimental data. Residues are reported in (c). All the curves are registered while heating the samples.

The effect of halloysite on the crystallinity of the paraffin was investigating by micro differential scanning calorimetry (μ-DSC). Thermograms of pure wax, wax/HNTs 0.05% w/w, and wax/HNTs 0.5% w/w Pickering emulsions are reported in Figure 7a.

As a general result, we observed an endothermic signal that can be attributed to the melting of wax.^77^ In particular, pure paraffin has two peaks related to two different phase changes and due to the existence of a metastable intermediate solid phase. During melting, the first phase change peak occurs at 32.5 °C, corresponding to the solid–metastable solid phase transition of the pure paraffin. The second peak occurs at 47.5 °C, corresponding to the metastable solid–liquid phase change. It should be noted that the transition temperatures are not altered by the addition of halloysite in the wax/HNTs Pickering emulsions. Furthermore, the peaks integration provides the enthalpy (Δ*H*_m_) of the paraffin melting process, which is expressed as joule per gram of wax. It is clear, by the observation of the curves reported in Figure 7a, that the nanoclay entrapment onto the outer surface of wax induced a reduction of Δ*C_p_* and Δ*H*_m_, highlighting a modification of the thermodynamics of the melting phase transitions. In particular, the values of the enthalpy calculated by the integration of the full thermograms are reported in Table 1.

**Table 1:** Enthalpy of the Melting Process (Δ*H*_m_) and Ratios between the Areas of the Curves Representing the Solid–Metastable Solid (Δ*H*_S–M_) and the Metastable Solid–Liquid (Δ*H*_M–L_) Transitions in the DSC Thermograms, Derived from Deconvolution and Nonlinear Fitting

sample	Δ*H*_m_ (J g^–1^)	Δ*H*_M–L_/Δ*H*_S–M_
pure wax	168.3	2.41
wax/HNTs 0.05% w/w	104.4	2.52
wax/HNTs 0.5% w/w	137.3	2.63

The decrease of Δ*H*_m_ after the addition of halloysite is due to the presence of the nanotubes, which partially destroy the crystallinity of paraffin, as observed for both beeswax and mineral wax.^60^ These results are consistent with the interaction arising between HNTs and the organic moiety, since the presence of attractive forces between nanoparticles and polymeric chains is responsible for a loss of the crystallinity of the polymer if it is anchored to the nanoparticles surface, as reported in the literature.^78^

With the aim to have more precise insights into the thermodynamics of the melting process, the deconvolution of the DSC curves was carried out in order to evaluate the two distinct contributions, namely the “solid–metastable solid” and the “metastable solid–liquid” phase transitions. Figure 7b reports the result of data analysis for the wax/HNTs 0.05% w/w Pickering emulsions, as an example. By integration of the two separate curves (the blue and green lines in the figure), it is possible to estimate the enthalpies for the solid–metastable solid (Δ*H*_S–M_) and for the metastable solid–liquid (Δ*H*_M–L_) transitions, respectively. Table 1 reports the ratio between Δ*H*_M–L_ and Δ*H*_S–M_ for the three investigated samples. It is noteworthy that the Δ*H*_M–L_/Δ*H*_S–M_ values increase with the concentration of halloysite nanotubes, thus meaning that the contribution of the solid–metastable solid phase change is lower. This is most likely due to the effect of HNTs. Since a reduction of enthalpy clearly means that a specific transition is favored, the solid–metastable solid transition of wax is favored after its interaction with the clay. To explain this we can admit that paraffin in the Pickering emulsions partially is at its metastable solid state, even below the transition temperature, as a consequence of interactions with halloysite in the external shell. Therefore, the endothermic contribution for this particular transition is reduced, and its enthalpy is lower compared to the massive bulk system.

### 3.3 Treatment of Archaeological Woods by Wax/HNTs Pickering Emulsions

The archeological waterlogged wood was a *Pinus pinaster* with a water content of 82 wt %. Based on cell wall density (1.38 g cm^–3^) and water density (0.997 g cm^–3^), this value corresponds to a porosity of 86.3% by volume (calculation details in the Supporting Information). Upon drying, the wood sample undergoes a shrinking and a consequent volume reduction up to 40%.

**Figure 8:**
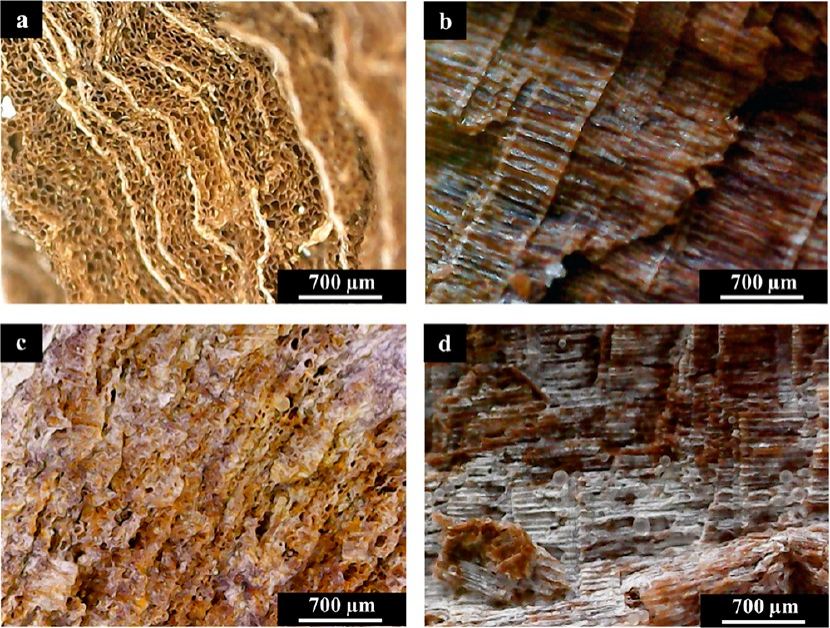
Optical micrographs of the transversal section and radial section of untreated wood (a, b) and consolidated wood using the wax/HNTs 0.5% w/w Pickering emulsions (c, d).

Therefore, wax/HNTs Pickering emulsions were investigated as consolidants for waterlogged archeological woods using the impregnation procedure. It is clear that one of the most important features of the proposed consolidation protocol is its ecofriendliness. Indeed, this method can be considered environmentally safe being that water was used as a unique immersion solvent for wood impregnation. Moreover, it overcomes some limitations regarding the possibility to be scaled up and employed for samples with large dimensions. To assess the protocol efficiency, we first focused on the morphology of untreated and treated wood samples, after impregnation into the wax/HNTs Pickering emulsions system. Optical micrographs are reported in Figure 8.

It is possible to observe the empty channels in the transversal section of the untreated wood sample (Figure 8a) as well as their regular alignment along the radial section (Figure 8b). It is worth to note that all the channels have the same average diameter (tens of micrometers), as expected for *Pinus pinaster*.^79^ More interestingly, after the impregnation procedure into the Pickering emulsions system was carried out, only a small portion of empty channels can be observed in the transversal section, most of them being coated and filled by the wax/HNTs microparticles (Figure 8c). Moreover, the consolidating material also appears on the surface of the wooden fibers, and some microparticles can be observed (Figure 8d).

Since the treatment of historical artifacts should preserve their structures without detrimental effects on their aspects, we conducted colorimetric analysis on the wooden samples before and after the impregnation. The color parameters in the lab scale are reported in Table S1 for all the investigated surfaces. We found that the total color difference (Δ*E*) between the untreated and the treated woods shows values ranging between 2.5 and 3.8, corresponding to just a noticeable difference by the human eye, as reported in the literature.^80,81^ As a consequence, the treatment has no critical side effects on the wood colorimetric features.

**Figure 9:**
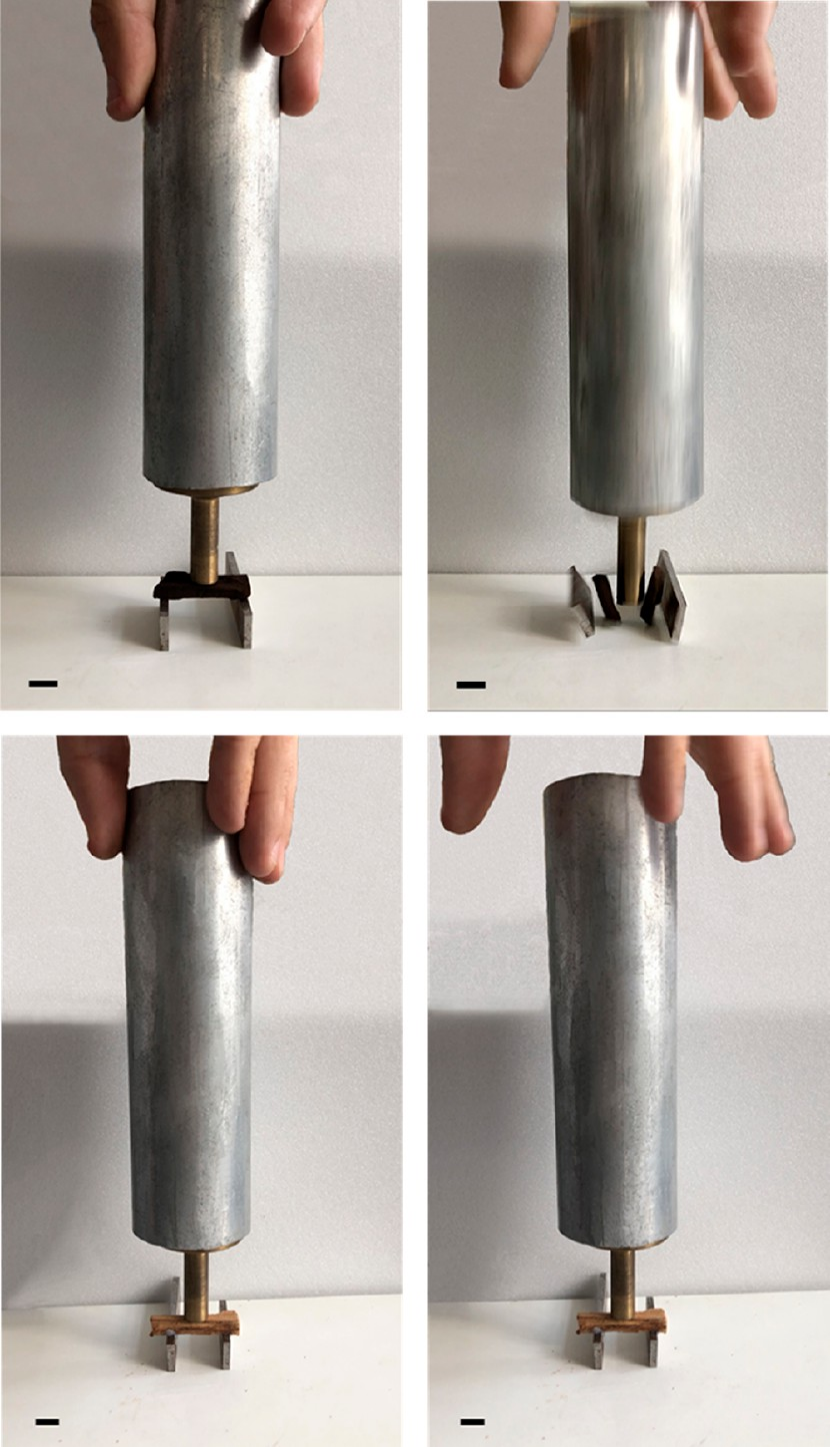
Optical photos of the waterlogged archeological wood before (up) and after (down) consolidation with wax/HNTs 0.5% w/w Pickering emulsions. A mass object of 0.960 kg is placed on the top of the samples. In these photos, the untreated wood is still wet. The scale bar is 1 cm.

Besides, the optical photographs in Figure 9 show that the consolidated wood sample exhibited robustness and mechanical resistance from the macroscopic point of view after its treatment. Contrarily, the untreated sample is very fragile, and it breaks very easily.

**Figure 10:**
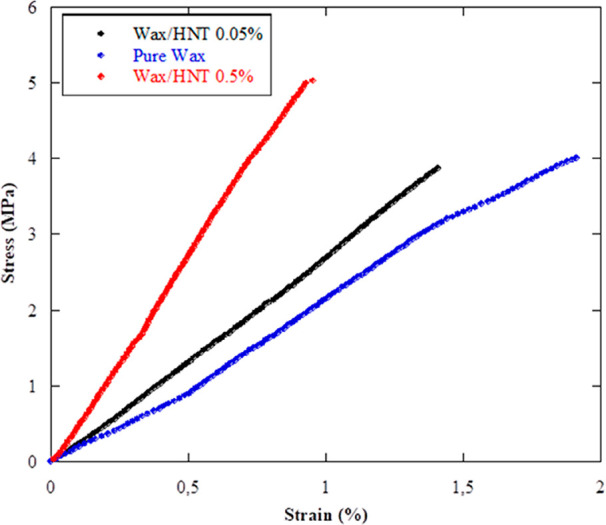
Stress vs strain curves for archeological wood consolidated with pure wax, wax/HNTs 0.05% w/w, and wax/HNTs 0.5% w/w Pickering emulsions.

The consolidation efficiency was tested by performing dynamic mechanical experiments (DMA). In particular, the mechanical properties of the treated samples were evaluated from the stress vs strains curves (Figure 10) to estimate important parameters such as Young’s modulus, stress at breaking, and elongation at breaking, which are summarized in Table 2. It should be noted that the untreated wood sample was not tested because of its high fragility.

**Table 2:** Young’s Modulus, Stress at Breaking, and Elongation at Breaking for Treated Waterlogged Archeological Woods for All the Tested Consolidant Materials^a^

	elastic modulus/MPa	stress at breaking point/MPa	elongation at breaking point/%
pure wax	214	4.0	1.9
wax/HNTs 0.05% w/w	282	3.9	1.4
wax/HNTs 0.5% w/w	549	5.1	0.9

a The relative error is 2%.

A strong enhancement of the elastic modulus of the treated wooden samples after consolidation with the wax/HNTs Pickering emulsions was observed, reaching a maximum when the concentration of the nanoclay was 0.5% w/w. Hence, the stiffness of the archeological wood was strongly improved. Also, the stress at the breaking point showed similar trends with the highest value for the wax/HNTs 0.5% w/w Pickering emulsions treated sample. Contrary to it, the elongation at the breaking point showed a relevant reduction, with a loss of ca. 50% of the elongation capability. Moreover, the aspect of the specimen was not altered upon drying, and its volume reduction was lower than 5%, confirming the presence of wax/HNTs microparticles inside the channels. In light of these observations, we can conclude that, due to their dimensions, the wax/HNTs Pickering emulsions can access the wooden structure by coating and filling the empty volume within the channels, as observed by optical microscopy and morphological characterization. Herein, the consolidating material plays a crucial role in avoiding the collapse of the wooden structure, thus improving the rigidity of the waterlogged wood and its mechanical properties. It should be noted that the mechanical performance of the consolidated wooden samples is of the same order of magnitude as that of sound wood from the same species.

## 4 Conclusions

Pickering emulsions composed of paraffin as inner core and halloysite clay nanotubes as outer shell were obtained by exploiting the thermal features of wax (i.e., its melting properties) and by varying the amounts of inorganic solid during the preparation procedure. Herein, HNTs are disposed at the wax/water interface, and they are partially entrapped into the organic moiety after cooling the dispersions, as observed by SEM analysis. Statistical analysis of the micrographs allowed the assessment of the dependence of the Pickering emulsions dimensions on the concentration of clay used for their preparation. For instance, the overall size presents lower values as the amount of halloysite becomes higher. Besides the effects on the diameter of the Pickering emulsions, the presence of the nanotubes also plays a major role in the variation of the melting thermodynamics of wax. Despite the fact that the temperatures of the solid to metastable solid and metastable solid to liquid transitions typical of wax did not change as a consequence of the addition of halloysite, the enthalpy of the paraffin melting process (Δ*H*_m_) was reduced. In particular, the decrease of Δ*H*_m_ is due to the presence of the nanotubes, which partially destroy the crystallinity of paraffin. Moreover, the deconvolution of the μ-DSC thermograms allowed the differentiation of the two distinct phase transitions, highlighting that the contribution of the solid–metastable solid phase transition is lower when the concentration of halloysite increases. Hence, the clay already makes the paraffin wax a metastable solid to a certain extent. At this point, the prepared materials were used as consolidants for waterlogged historical woods. By optical microscopy and colorimetric analysis, we observed that the channels of the treated wood are filled with the microparticles without affecting the macroscopic aspect of the sample. After the treatment, an overall improvement of the mechanical performances was revealed. Both Young’s modulus and stress at breaking increased as a consequence of the major stiffness and rigidity after the treatment, whereas the elongation at break decreased due to the pores and channels filling with Pickering emulsions in the archeological structures. Most importantly, the key aspects of crucial importance in the proposed consolidation protocol are the absence of side effects to its use and the unique ecocompatibility and environmental friendliness. Indeed water is the only solvent used in the whole method, which thus shows great potential to be scaled up. In conclusion, this study is encouraging for designing a green protocol for the durable preservation of waterlogged archeological woods by using biocompatible and ecosustainable Pickering emulsions based on paraffin wax and halloysite nanotubes, and interesting perspectives are opened and can be investigated for the treatment of shipwrecks of large dimensions. Besides, since other polymeric species that possess a similar melting temperature could be in principle employed for the same purpose, the proposed protocol is also versatile as it could be extended to the use of other materials.

## Supporting Information Available

Details on the optimization of the Pickering emulsion preparation protocol; details on the recyclability of wax/HNTs Pickering emulsions; calculation details of the water content and porosity of archeological wood; colorimetric parameters (PDF)
