# Supplementary material for: Pickering Emulsions Based on Wax and Halloysite Nanotubes: An Ecofriendly Protocol for the Treatment of Archeological Woods
Source: ACS Appl Mater Interfaces. 2020 Dec 30;13(1):1651–61. doi: 10.1021/acsami.0c20443 (PMC8021222; doi:10.1021/acsami.0c20443)
Supplement: Supplementary file 1 — am0c20443_si_001.pdf [file am0c20443_si_001.pdf]

SUPPORTING INFORMATION

Pickering Emulsions based on Wax and Halloysite Nanotubes: an Eco-Friendly Protocol for the Treatment of Archeological Woods.

Lorenzo Lisuzzo[ab*], Theodore Hueckel[a], Giuseppe Cavallaro[b], Stefano Sacanna[a], Giuseppe Lazzara[b],

[a] Molecular Design Institute, Department of Chemistry, New York University, 29 Washington Place, New York, New York 10003, United States

[b] Department of Physics and Chemistry, University of Palermo, Viale delle Scienze, pad. 17, Palermo 90128, Italy

[*]lorenzo.lisuzzo@unipa.it

Preparation of Wax/HNTs Pickering emulsions

Scanning Electron Microscopy allowed to assess the effectiveness of each phase in the preparation protocol of the hybrid systems. Indeed, both the yield and the quality of the Pickering emulsions is very low when one of the crucial steps is missing. As an example, SEM images of the system without 30 min equilibration at 80 °C and ice bath quenching are reported.

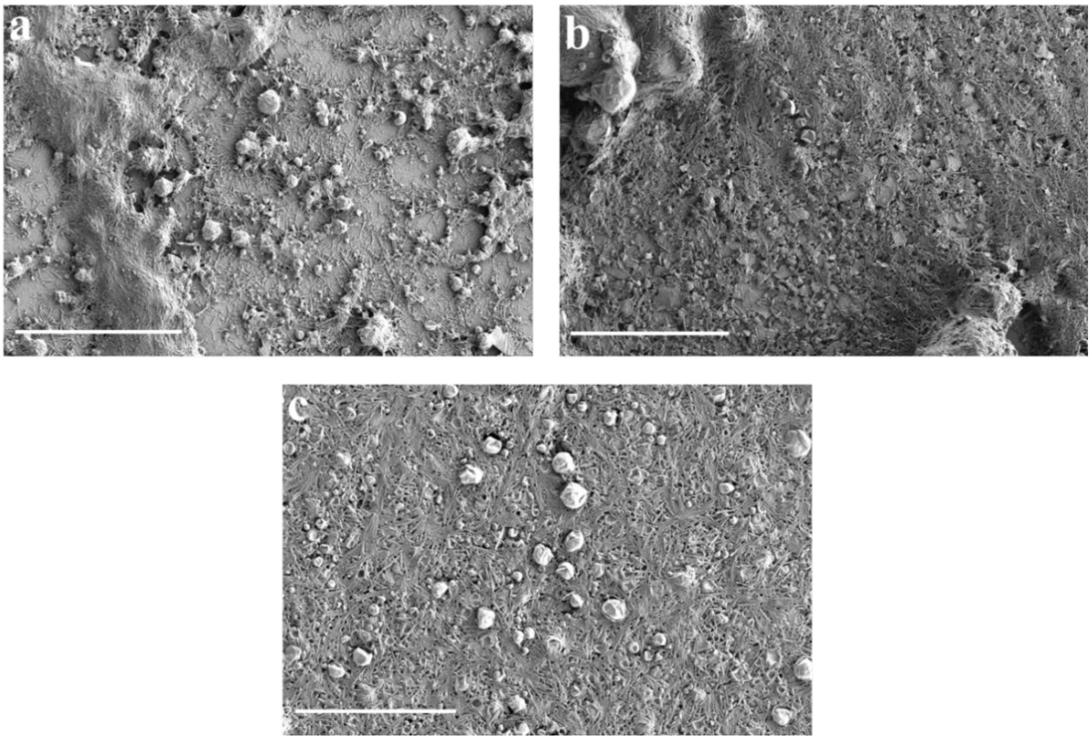

Figure S1. SEM images of Wax/HNTs systems with (a) 0.1 % w/w, (b) 0.5 % w/w and (c) 1 % w/w Halloysite concentrations before optimizing the preparation protocol. Scale bars are 60 µm.

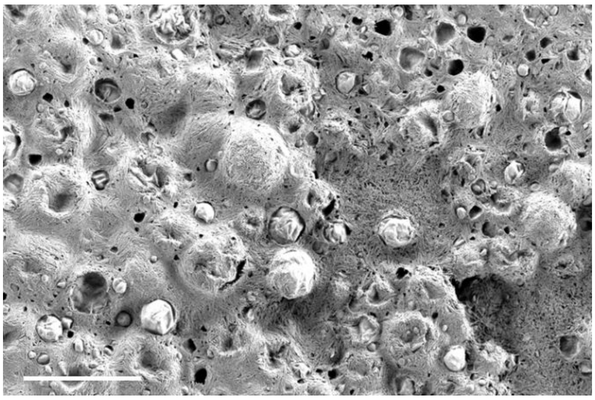

Figure S2. SEM image of Wax/HNTs emulsions with 1 %w/w halloysite concentration. Scale bar is 60 μm.

Recyclability of Wax/HNTs Pickering emulsions

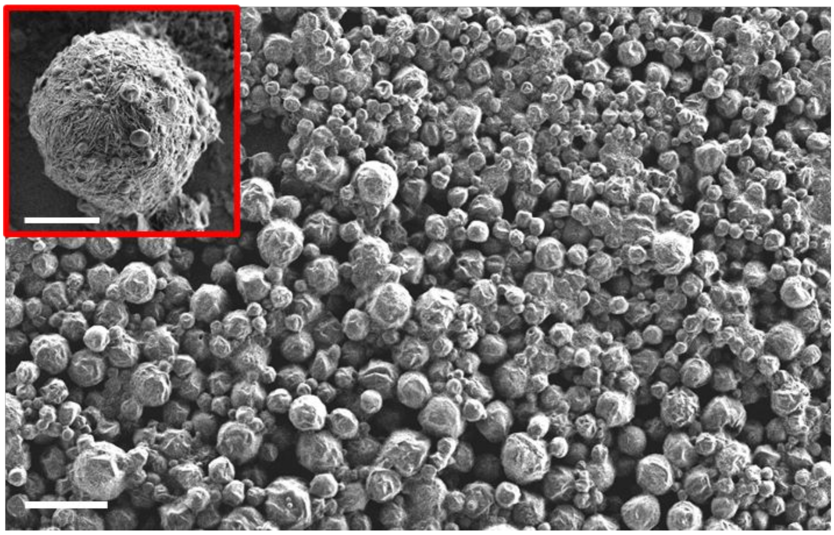

Figure S3. SEM image of the wax/HNTs 0.5% w/w Pickering emulsions after heating up to the melting of wax and treating again according to the preparation protocol. Scale bars are 60 μm for the image and 20 μm for the inset.

Water Content and Porosity of Wood

Based on gravimetric data, water content (WC%) and porosity (Z%) of wood were calculated as reported below:

- WC% = 100 x (mass of water in wet wood sample/mass of wet wood sample)
- Z% = 100 x (volume of water)/(volume of water + volume of dry wood)

Colorimetric Analysis

Table S1. Colour Parameters of untreated wood and consolidated wood using the Wax/HNTs 0.5% w/w Pickering emulsions system.[a]

	**L***	**a***	**b***	**ΔE***
Untreated wood_1	38.326	10.478	16.246	
Consolidated wood_1	38.624	8.657	13.205	3.6
Untreated wood_2	39.715	11.503	17.332	
Consolidated wood_2	38.788	10.790	13.754	3.8
Untreated wood_3	38.538	11.080	16.889	
Consolidated wood_3	38.612	10.230	14.497	2.5

[a]Control: White Paper standard, L*=92.278, a*=-0.018, b*=1.905
